# mRNA expression of genes involved in inflammation and haemostasis in equine fibroblast-like synoviocytes following exposure to lipopolysaccharide, fibrinogen and thrombin

**DOI:** 10.1186/s12917-015-0448-z

**Published:** 2015-06-27

**Authors:** Stine Mandrup Andreassen, Lise C. Berg, Søren Saxmose Nielsen, Annemarie T. Kristensen, Stine Jacobsen

**Affiliations:** Department of Large Animal Sciences, Medicine and Surgery group, University of Copenhagen, Højbakkegård allé 5, DK-2630 Tåstrup, Denmark; Department of Veterinary Clinical and Animal Sciences, University of Copenhagen, Dyrlægevej 16, DK-1870 Frederiksberg C, Denmark; Department of Large Animal Sciences, University of Copenhagen, Grønnegårdsvej 8, DK-1870 Frederiksberg C, Denmark

**Keywords:** Equine, Fibroblast-like synoviocytes, Fibrinogen, Lipopolysaccharide, Thrombin, mRNA expression, Inflammation, Coagulation, Fibrinolysis, Haemostasis

## Abstract

**Background:**

Studies in humans have shown that haemostatic and inflammatory pathways both play important roles in the pathogenesis of joint disease. The aim of this study was to assess mRNA expression of haemostatic and inflammatory factors in cultured equine fibroblast-like synoviocytes exposed to lipopolysaccharide (LPS), fibrinogen and thrombin. Synovial membranes were collected from metacarpo-phalangeal joints of 6 skeletally mature horses euthanized for non-orthopaedic reasons. Passage 4 fibroblast-like synoviocytes were left non-treated or treated with either 0.1 μg/ml LPS, 5 mg/ml fibrinogen or 5 U/ml thrombin and harvested at time points 0, 6, 24 and 48 h. mRNA expression of serum amyloid A (SAA), interleukin-6 (IL-6), monocyte chemotactic protein 1 (MCP-1), tissue factor (TF), plasminogen activator inhibitor 1 (PAI-1), urokinase plasminogen activator (uPA), vascular endothelial growth factor (VEGF) and protease activator receptor 1 (PAR-1) was assessed using quantitative real time reverse transcriptase PCR.

**Results:**

LPS caused a significant increase in mRNA expression of SAA, IL-6, MCP-1 and uPA, and a decrease in TF, PAI-1 and PAR-1 when compared to non-treated cells. Treatment with thrombin resulted in increased mRNA expression of SAA, IL-6, MCP-1 and PAI-1, and a decreased PAR-1 expression compared to non-treated cells. The fibrinogen-treated synoviocytes showed significantly increased mRNA expression of IL-6, MCP-1, TF and PAI-1, and decreased PAR-1 expression compared to non-treated cells.

**Conclusion:**

LPS, fibrinogen and thrombin induced an increased gene expression of inflammatory markers in isolated equine fibroblast-like synoviocytes. LPS caused changes in gene expression promoting increased fibrinolysis, while fibrinogen and thrombin changed the gene expression resulting potentially in reduced fibrinolysis. Overall, it appeared that both inflammatory and haemostatic stimuli affected expression of genes involved in inflammatory and haemostatic pathways, supporting their importance in equine joint diseases.

## Background

Joint diseases such as osteoarthritis (OA), osteochondrosis dissecans and septic arthritis affect horses regularly and are common causes of lameness and reduced performance [1–3]. The pathogenesis of joint disease is complex, but an inflammatory reaction is a common characteristic and has been studied extensively in the horse [4–6]. Increased levels of inflammatory components such as leukocytes, serum amyloid A (SAA), interleukin (IL)-1β, IL-6, tumour necrosis factor α (TNF- α), and matrix metalloproteinases have been demonstrated in horses with naturally occurring and experimentally induced septic arthritis and OA [4, 7–9]. Fibroblast-like synoviocytes are known to contribute to progression of joint disease [10], and in the pathogenesis of OA it is recognised that fibroblast-like synoviocytes participate by producing proinflammatory cytokines and cartilage-degrading mediators [11–13]. Equine septic arthritis and rheumatoid arthritis (RA) in humans are both characterized as severe inflammatory conditions showing similar pathologic occurrences such as intra-articular cell recruitment and synoviocyte driven cytokine release [4, 6, 14–16].

Recent research in human joint disease has indicated that haemostatic pathways also play an important part in the pathogenesis of joint disease, and that activation of inflammation and haemostasis are closely related [17]. Haemostatic components are not only present in the initial stages of an intra-articular inflammatory event, it also seems that the two systems interact throughout the disease process [18]. Studies in humans and rodents have shown that both coagulation and fibrinolysis are activated intra-articularly in RA and OA [15, 19].

To our knowledge the potential co-occurrence of intra-articular expression of inflammatory and haemostasis markers in the horse has not previously been investigated. Lipopolysaccharide (LPS) has been described to elicit both in vivo and in vitro arthritic inflammatory responses [7, 16, 20]. Thrombin, a key proteinase in the process of fibrin formation, has been reported to be elevated in synovial fluid from humans with OA and RA [21], and when synoviocytes derived from patients with RA were exposed to thrombin, their production of IL-6 increased [14, 22]. Fibrinogen, a large coagulant protein synthesized by hepatocytes and normally absent or present in very low concentration in non-diseased synovial fluid, has been detected in elevated concentrations in synovial fluid from human OA patients [23]. In human synovial fibroblasts and pancreatic stellate cells fibrinogen has been found to induce inflammatory reactions [24, 25]. Fibrin(ogen) may regulate progression of inflammation in joint disease and has the potential to maintain inflammation [18, 24]. It has been reported that fibrinolysis is needed to resolve joint inflammation [15]. Fibrin aggregates were found in synovial fluid in rabbits within 24 h of antigen-induced arthritis [26], and in horses recently published papers have shown for the first time that d-dimer, an end-product of fibrinolysis, is present in increased concentrations in synovial fluid in joint disease [27, 28]. This indicates that, along with inflammation, haemostasis is activated and may play a role in the pathogenesis of joint disease in horses and other species.

The overall aim of this study was to further our understanding of the events taking place in the early stages of joint disease by studying effects of inflammatory and haemostatic stimuli on gene expression in cultured fibroblast-like synoviocytes. The specific objectives were to investigate the effects of LPS, fibrinogen and thrombin on the expression of genes representing acute inflammation (IL-6, SAA, monocyte chemotactic protein-1 [MCP-1]), initiation of haemostasis (tissue factor [TF]), fibrinolysis (urokinase plasminogen activator [uPA], plasminogen activator inhibitor-1 [PAI-1]), angiogenesis (vascular endothelial growth factor [VEGF]) and thrombin-binding receptor (proteinase activated receptor-1 [PAR-1]) in equine fibroblast-like synoviocytes.

## Results

**Fig. 1:**
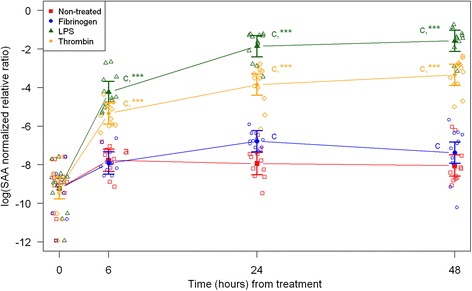
mRNA expression levels of serum amyloid A (SAA) from equine fibroblast-like synoviocytes shown as log-transformed GAPDH-normalized relative ratios. Error bars show SE centered on the mean. Raw data are plotted with open symbols. Asterisks designate significant difference from non-treated within a time point (* = *p* < 0.05; ** = *p* < 0.01 and *** = *p* < 0.001). Superscript letters designate significant differences from time point 0 within a treatment (a = *p* < 0.05; b = p < 0.01 and c = p < 0.001). Treatments of the equine synovial fibroblast are coloured (Non-treated = red; fibrinogen = blue; LPS = green; thrombin = orange)

**Fig. 2:**
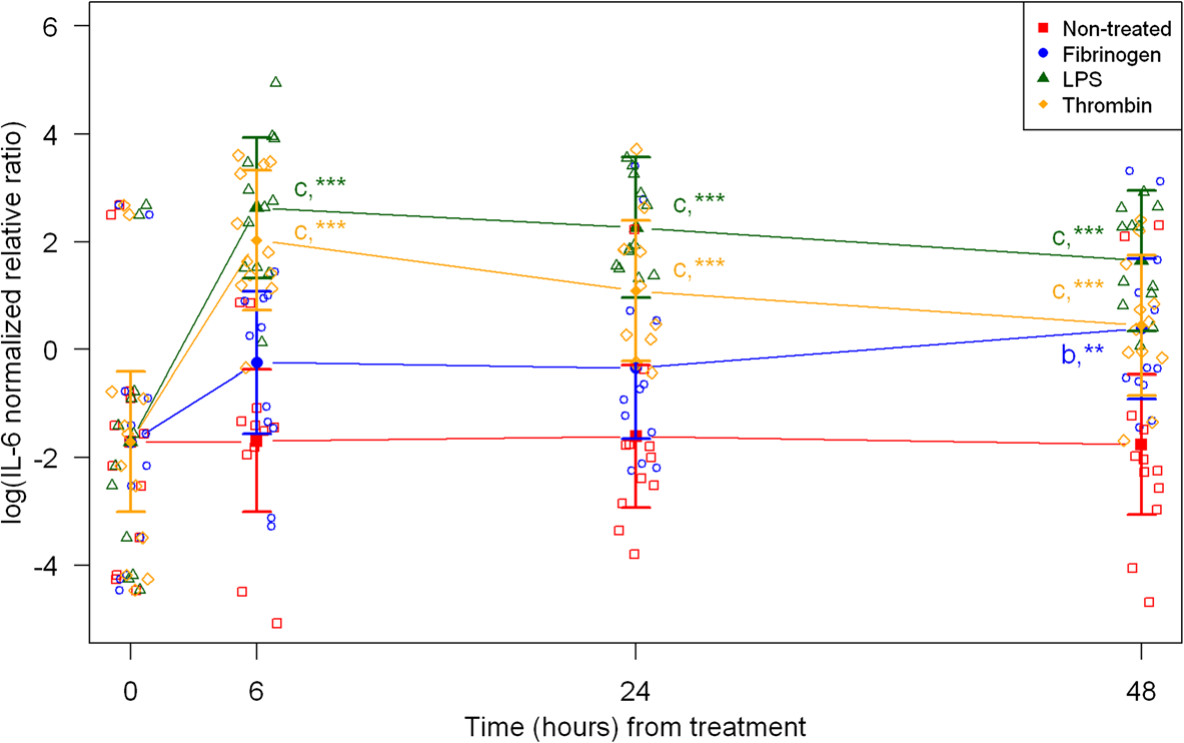
mRNA expression levels of interleukin-6 (IL-6) from equine fibroblast-like synoviocytes shown as log-transformed GAPDH-normalized relative ratios. Error bars show SE centered on the mean. Raw data are plotted with open symbols. Asterisks designate significant difference from non-treated within a time point (* = p < 0.05; ** = p < 0.01 and *** = p < 0.001). Superscript letters designate significant differences from time point 0 within a treatment (a = p < 0.05; b = p < 0.01 and c = p < 0.001). Treatments of the equine synovial fibroblast are coloured (Non-treated = red; fibrinogen = blue; LPS = green; thrombin = orange)

**Fig. 3:**
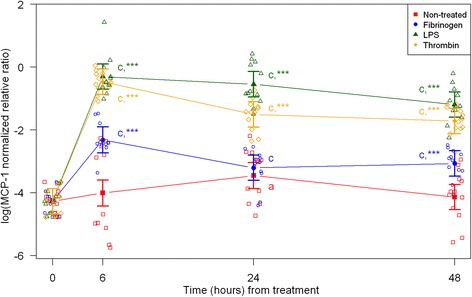
mRNA expression levels of monocyte chemotactic protein-1 (MCP-1) from equine fibroblast-like synoviocytes shown as log-transformed GAPDH-normalized relative ratios. Error bars show SE centered on the mean. Raw data are plotted with open symbols. Asterisks designate significant difference from non-treated within a time point (* = p < 0.05; ** = p < 0.01 and *** = p < 0.001). Superscript letters designate significant differences from time point 0 within a treatment (a = p < 0.05; b = p < 0.01 and c = p < 0.001). Treatments of the equine synovial fibroblast are coloured (Non-treated = red; fibrinogen = blue; LPS = green; thrombin = orange)

**Fig. 4:**
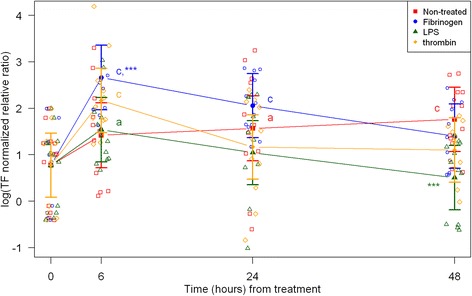
mRNA expression levels of tissue factor (TF) from equine fibroblast-like synoviocytes shown as log-transformed GAPDH-normalized relative ratios. Error bars show SE centered on the mean. Raw data are plotted with open symbols. Asterisks designate significant difference from non-treated within a time point (* = p < 0.05; ** = p < 0.01 and *** = p < 0.001). Superscript letters designate significant differences from time point 0 within a treatment (a = p < 0.05; b = p < 0.01 and c = p < 0.001). Treatments of the equine synovial fibroblast are coloured (Non-treated = red; fibrinogen = blue; LPS = green; thrombin = orange)

**Fig. 5:**
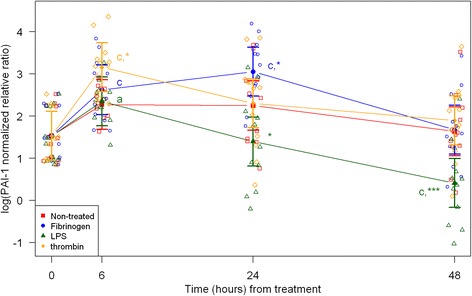
mRNA expression levels of plasminogen activator inhibitor-1 (PAI-1) from equine fibroblast-like synoviocytes shown as log-transformed GAPDH-normalized relative ratios. Error bars show SE centered on the mean. Raw data are plotted with open symbols. Asterisks designate significant difference from non-treated within a time point (* = p < 0.05; ** = p < 0.01 and *** = p < 0.001). Superscript letters designate significant differences from time point 0 within a treatment (a = p < 0.05; b = p < 0.01 and c = p < 0.001). Treatments of the equine synovial fibroblast are coloured (Non-treated = red; fibrinogen = blue; LPS = green; thrombin = orange)

**Fig. 6:**
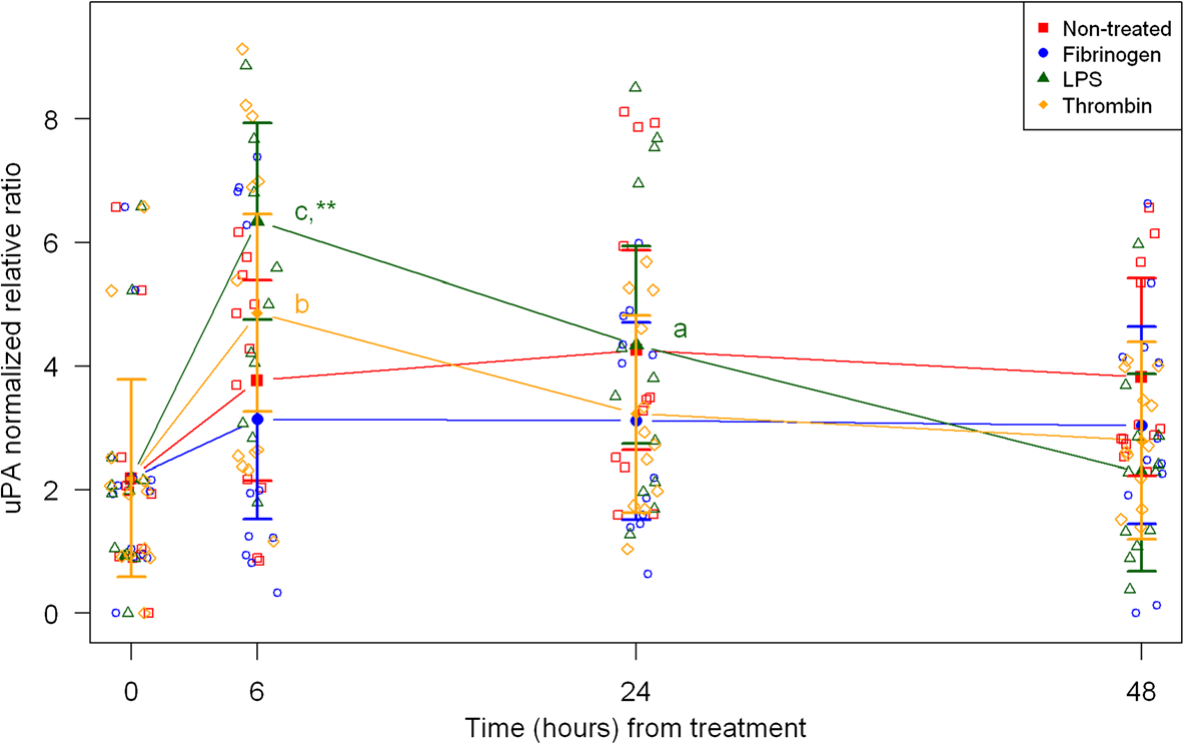
mRNA expression levels of urokinase plasminogen activator (uPA) from equine fibroblast-like synoviocytes shown as GAPDH-normalized relative ratios. Error bars show SE centered on the mean. Raw data are plotted with open symbols. Asterisks designate significant difference from non-treated within a time point (* = p < 0.05; ** = p < 0.01 and *** = p < 0.001). Superscript letters designate significant differences from time point 0 within a treatment (a = p < 0.05; b = p < 0.01 and c = p < 0.001). Treatments of the equine synovial fibroblast are coloured (Non-treated = red; fibrinogen = blue; LPS = green; thrombin = orange)

**Fig. 7:**
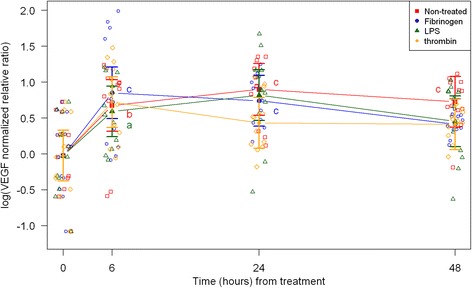
mRNA expression levels of vascular endothelial growth factor (VEGF) from equine fibroblast-like synoviocytes shown as log-transformed GAPDH-normalized relative ratios. Error bars show SE centered on the mean. Raw data are plotted with open symbols. Asterisks designate significant difference from non-treated within a time point (* = p < 0.05; ** = p < 0.01 and *** = p < 0.001). Superscript letters designate significant differences from time point 0 within a treatment (a = p < 0.05; b = p < 0.01 and c = p < 0.001). Treatments of the equine synovial fibroblast are coloured (Non-treated = red; fibrinogen = blue; LPS = green; thrombin = orange)

**Fig. 8:**
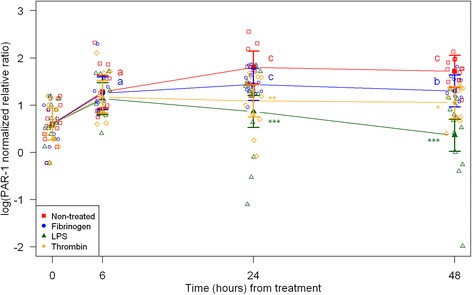
mRNA expression levels of proteinase activated receptor 1 (PAR-1) from equine fibroblast-like synoviocytes shown as square root-transformed GAPDH-normalized relative ratios. Error bars show SE centered on the mean. Raw data are plotted with open symbols. Asterisks designate significant difference from non-treated within a time point (* = p < 0.05; ** = p < 0.01 and *** = p < 0.001). Superscript letters designate significant differences from time point 0 within a treatment (a = p < 0.05; b = p < 0.01 and c = p < 0.001). Treatments of the equine synovial fibroblast are coloured (Non-treated = red; fibrinogen = blue; LPS = green; thrombin = orange)

Cell morphology became increasingly more uniform across passages. In the first passage, round and amorphous cells without pseudopods—resembling macrophage-like synoviocytes (type A cells)—were occasionally observed. In the third and fourth passage, cells were homogenous and had large pseudopods, characteristics of fibroblast-like synoviocytes (type B cells) [29, 30]. After thawing and at every passage cell viability was assessed to > 95 % (data not shown). Following, results are described according to treatments, and shown in figures according to outcome parameter (Figs. 1, 2, and 3: acute inflammation (SAA, IL-6, MCP-1); Fig. 4: initiation of haemostasis (TF); Figs. 5 and 6: fibrinolysis (uPA, PAI-1); Fig. 7: angiogenesis (VEGF) and Fig. 8: thrombin-binding receptor (PAR-1)).

### Non-treated synoviocytes

For the non-treated synoviocytes there were no statistically significant changes in expression of IL-6, PAI-1 and uPA over time (Figs. 2, 5, 6). The expression of VEGF and PAR-1 increased significantly over time with expression levels at every time point being significantly higher than expression levels at 0 h (Figs. 7 and 8). Expression levels of SAA, MCP-1 and TF were significantly higher than expression levels at 0 h on one or two time points (SAA: 6 h, MCP-1: 24 h, TF: 24 and 48 h) (Figs. 1, 3 and 4).

### Treatment with LPS

In synoviocytes treated with LPS mRNA expression of SAA, IL-6 and MCP-1 was significantly higher than expression at 0 h at every time point (6, 24, and 48 h). Also, their expression levels were significantly higher than expression levels in the corresponding non-treated control at every time point (Figs. 1, 2 and 3). Expression of VEGF showed statistically significant increases over time compared to time point 0, but no differences were detected between the LPS stimulated synoviocytes and the non-treated controls (Fig. 7). Expression of TF, PAI-1 and uPA showed intermittent significant increases relative to 0 h after LPS treatment (Figs. 4, and 6). TF, PAI-1 and PAR-1 mRNA expression levels were significantly lower than expression levels in the corresponding non-treated control at 24 and/or 48 h in response to LPS treatment (Figs. 4, 5 and 8). uPA mRNA expression levels increased significantly at 6 h after treatment compared to non-treated control (Fig. 6).

### Treatment with fibrinogen

In fibrinogen-treated synoviocytes, mRNA expression of SAA increased significantly over time, but expression levels were not significantly different from the corresponding non-treated control (Fig. 1). mRNA expression of IL-6, MCP-1, TF and PAI-1 in fibrinogen treated synoviocytes was significantly increased at one or more time points relative to 0 h, and their mRNA expression levels were significantly higher than the corresponding non-treated control at one or two time points after treatment (IL-6: 48 h, MCP-1: 6 and 48 h, TF: 6 h and PAI-1: 24 h; Figs. 2, 3, 4 and 5). mRNA expression of uPA, VEGF and PAR-1 did not change significantly in response to the fibrinogen treatment compared to non-treated control. VEGF and PAR-1 did show significant increases compared to time point 0 at two or three time points (Figs. 6, 7 and 8).

### Treatment with thrombin

Thrombin treatment of equine synoviocytes resulted in significantly higher mRNA expression of SAA, IL-6 and MCP-1 compared to non-treated controls and to expression at 0 h at every time point (6, 24 and 48 h; Figs. 1, 2 and 3). mRNA expression of PAI-1 was significantly increased at 6 h after thrombin treatment compared to non-treated controls (Fig. 5). mRNA expression of PAR-1 was significantly decreased at 24 and 48 h after treatment compared to non-treated controls (Fig. 8). At 6 h after thrombin treatment mRNA expression of TF, PAI-1, uPA, VEGF and PAR-1 were significantly increased compared to time point 0 (Figs. 4, 5 and 8).

## Discussion

The results of the study showed that LPS exerted its well-described proinflammatory effects [31] in isolated equine synoviocytes by increasing expression of SAA, IL-6 and MCP-1. Moreover, LPS induced alteration of expression of factors affecting the fibrinolytic potential of the synoviocytes (up-regulation of uPA and down-regulating PAI-1) and caused a decrease in mRNA expression of TF. Thrombin treatment also induced inflammatory changes by increased expression of SAA, IL-6 and MCP-1, but decreased fibrinolysis by an early increase in PAI-1. Fibrinogen exposure also caused an increase in expression of inflammatory markers (IL-6 and MCP-1). In contrast to LPS, fibrinogen induced increased mRNA expression of TF and PAI-1 in the synoviocytes—changes that have the potential to increase extravascular coagulation (TF) and inhibit fibrinolysis (PAI-1). All three treatments decreased the mRNA expression of PAR-1.

A common characteristic of several joint conditions is inflammation, and studies have shown its importance in development and healing of disease processes [15, 17, 18]. Lately haemostatic reactions have obtained interest, since upregulation of factors involved in coagulation and fibrinolytic pathways has been shown to occur intra-articularly in mice with antigen-induced arthritis [19] and in humans with naturally occurring arthritis [15, 23]. It thus seems, just as in systemic reactions, that both inflammatory and haemostatic pathways are activated and likely interlinked in the joint cavity [17, 18]. One link between inflammation and haemostasis is fibrinogen, which is an acute phase protein upregulated during inflammation, but also serves a crucial role in haemostasis. Fibrinogen has been shown to serve as inducer and regulator of inflammatory reactions in joint diseases in humans and rodents [17, 18, 24, 32]. Thrombin is another link between inflammation and haemostasis. While thrombin has traditionally been thought of as a procoagulant through its induction of fibrin formation, it has also been shown to be a potent inducer of production of inflammatory biomolecules such as MCP-1 and IL-6 and activator of leukocytes [14, 33]. In horses, inflammatory changes in joints with spontaneous or experimentally-induced arthritis are well-described [20, 34, 35]. In contrast, only few studies have attempted to assess haemostatic factors intra-articularly in horses. Two recent studies have described increases in d-dimer in synovial fluid of horses with joint disease [28, 36], indicating that fibrinolysis is active in the intra-articular compartment of horses with joint inflammation. It is thus possible that the cross-talk between inflammation and haemostasis suggested as a hallmark of human joint disease [37, 38] may also be at play in horses.

LPS is a well-known inflammatory inducer, and it was therefore not surprising that it caused an up-regulation of mRNA expression of the inflammatory markers SAA and IL-6 starting at 6 h and continuing till the end of the study (48 h). Similar results have been found in other in vitro studies showing LPS-induced increases in SAA mRNA expression in chicken synovial fibroblast [30] and in IL-6 mRNA expression in equine and human synoviocytes [14, 16]. LPS exposure also caused the equine synoviocytes to express MCP-1 mRNA. While this to our knowledge is the first time that MCP-1 is described in horses, similar results have been found in IL-1β-treated human synoviocytes [39] and in mice with haemophilic synovitis [40]. MCP-1 serves to recruit mononuclear cells to the joint compartment [39–41], and MCP-1 has been suggested to correlate with the degree of inflammatory changes in human synovial membrane [42].

Thrombin stimulation caused significant increases in mRNA expressions of the three inflammatory markers of this study (SAA, IL-6 and MCP-1) in the synoviocytes, resembling the response observed after LPS stimulation. Thrombin-induced IL-6 production has previously been shown in human synovial fibroblasts [14], and MCP-1 induction by thrombin has been shown in endothelial cells [43] and MCP-1 was found elevated in synovial fluid from haemophilic mice [40]. Proinflammatory effects of thrombin have thus been demonstrated across species and tissues.

The inflammation-inducing potential of fibrinogen is less well described than that of LPS. In the present study, fibrinogen induced a significant increase in mRNA expression of IL-6 and MCP-1 after 48 and 6 h of stimulation, respectively. In species other than the horse, fibrinogen exposure has been described to cause inflammatory changes: increased IL-6 concentrations were found in culture supernatant from fibrinogen-treated human pancreatic stellate cells [25], and fibrinogen induced increases in MCP-1 expression was shown in endothelial cells of humans and pigs [44, 45]. In articular tissue, fibrinogen exposure has been shown to exert proinflammatory functions. Human synovial fibroblasts cultured in vitro in the presence of fibrinogen had increased expression of intercellular adhesion molecules (ICAM) and interleukin-8 (IL-8) [24]. By increasing expression of ICAM, IL-8, and MCP-1 fibrinogen may serve to attract and retain leukocytes intra-articularly, but at this stage the exact effects of fibrinogen in the intra-articular compartment are unknown.

Factors involved in haemostasis pathways were induced in equine synoviocytes after LPS, thrombin and fibrinogen exposure. Fibrinogen-treated synoviocytes showed increased mRNA expression of TF at 6 h and thereafter a gradual decline. In contrast, LPS-treatment decreased the mRNA expression of TF at 48 h. This is surprising and interesting, since LPS stimulation has been found to increase TF in equine peritoneal macrophages [46]. In vivo, a gradual increase in TF mRNA expression over 7 days has been demonstrated in mice with antigen-induced arthritis [19] and increased TF activities along with increased fibrinogen concentrations have been found in inflamed synovial fluid from human arthritis [23]. TF initiates cell based haemostasis [47] resulting in thrombin formation [48], but TF is also described as a link between inflammation and haemostasis [23]. TF has been suggested to play an active role in attraction of monocytes to the synovial membrane [49], and a significant correlation between synovial fluid TF concentrations and leukocyte counts has been demonstrated [15]. To further characterize and understand the synovial TF response in horses, the in vitro results presented here need to be corroborated by in vivo data.

The balance between uPA and PAI-1 determines the fibrinolytic activity in the joint [50, 51]. The LPS-treated equine synoviocytes in this study showed a significant increase in uPA mRNA expression at 6 h followed by a steady decline, and a gradual decrease in PAI-1 mRNA expression throughout the 48-h study period, which would increase fibrinolysis. In contrast, fibrinogen- and thrombin-treated synoviocytes did not show changes in uPA mRNA expression, while mRNA expression of PAI-1 increased significantly at 24 and 6 h, respectively. Taken together, this suggests that an inflammatory stimulus increases the fibrinolytic capacity of equine synoviocytes first by an uPA increase and then a PAI-1 decrease, whereas the haemostatic proteins fibrinogen and thrombin appear to inhibit the fibrinolytic capacity of equine synoviocytes by upregulation of PAI-1. In human synovial tissue obtained from patients with OA and RA both uPA and PAI-1 mRNA expression was increased [32], and in mice with antigen-induce arthritis uPA mRNA expression in synovial tissue quickly increased and peaked around 4 h, while PAI-1 was increased in the first 3 days after induction of arthritis [19]. These findings seemingly suggest that inflamed joints develop a pro- or hyper-coagulable state with tendency to produce and clot more fibrinogen [19]. If the same is true for the horse, the results of this study indicate that fibrinolytic pathways are intact in initial inflammatory arthritis, and when fibrinogen and thrombin accumulation occurs in the joint cavity, coagulation increases and fibrinolysis decreases, making the joint potentially vulnerable to fibrin deposition and its deleterious effects.

PAR-1, a trans-membrane receptor for thrombin and other proteinases [52], showed significantly lower expression at 24 and 48 h after all three treatments compared to the non-treated controls. LPS caused the most down-regulation of PAR-1 expression. PAR-1 knockout mice have shown less clinical symptoms in antigen-induced arthritis compared to wild type mice [53], and PAR-1 has also been demonstrated in human RA synovial fibroblasts [54, 55]. These results indicate that PAR-1 plays a role in joint disease processes [22]. Increased synovial fibroblast proliferation [54] and increased mRNA expression in synovial fibroblast of inflammatory cytokines IL-6, IL-8 and TNF-α have been shown to be mediated through PAR-1 [14, 56]. Whether inflammation up- or down-regulates expression of PAR-1 in articular tissues is not currently clear. Opposite to our results, PAR-1 has been found in increased levels in inflamed human muscle cells [57] and infected murine smooth muscle cell [58], and thrombin and LPS have been shown to induce PAR-1 mRNA expression in endothelial cells [59] and in rat astrocytes [60]. In accordance with the findings of our study, decreasing levels of PAR-1 mRNA has been found in ischemic rat brain [61] and after TNF-α stimulation of human endothelial cells [62]. Decreased PAR-1 expression in response to inflammatory and haemostatic stimuli might indicate a down-regulation of inflammatory pathways in equine synovial fibroblasts.

VEGF was the only marker investigated, whose expression pattern was not changed in response to any of the treatments. VEGF was initially expected to show increased expression pattern in the stimulated synoviocytes, as it has been demonstrated to have an angiogenic role in inflammatory joint diseases [63] and has been found in increased concentrations in synovial fluid of human OA and RA patients [21, 64]. The angiogenic effects and pro-inflammatory properties of VEGF in synovial inflammation can potentially lead to hyperplasia of the synovial membrane, pannus formation and development of osteoarthritis [65, 66]. Several cell types such as rat lung pericytes, human pancreatic stellate cells, human fibroblast, human and bovine endothelial cells have shown up-regulation of VEGF mRNA after LPS, thrombin or fibrinogen treatment [25, 67–69]. While studies in humans demonstrated increased VEGF concentrations in synovial fluid from RA patients [21, 64], a study by Salvi et al. [19] detected no change in VEGF expression in synovial tissue from antigen-induced arthritis in mice. Whether VEGF is consistently up-regulated in joint inflammation is thus not clear at this stage and warrants further study.

This is the first study evaluating the relationship between inflammation and haemostasis in equine fibroblast-like synoviocytes. Obviously, the single cell type with exposure to one inducing factor at a time cannot reflect the complexity of events taking place in an inflamed joint, and further studies are warranted before firm conclusions regarding the interplay between inflammatory and haemostatic pathways in equine joint disease may be drawn. However, the findings of our study help elucidate specific interactions between individual reactants, which are otherwise impossible to tease out of the complex microenvironment of the diseased joint.

In the present study, passage-4 equine synovial fibroblasts were used. It is well documented that at this passage the cell population is homogenous, because the culture environment does not favour the macrophage-like synoviocytes [29, 30]. It is clear from the results of the present study that it is important to include non-treated cell cultures at all investigated time points to control for culture-induced effects on mRNA expression of the chosen markers unrelated to the tested treatments. The non-treated cultures showed significant increases in mRNA expression of SAA, MCP-1, TF, VEGF and PAR-1 over time, showing that the culture environment affected the synoviocytes.

## Conclusion

In conclusion, the results of this study indicate that in equine fibroblast-like synoviocytes, inflammatory and haemostatic pathways are at play simultaneously and affect each other. All three treatments (LPS, fibrinogen and thrombin) increased gene expression of inflammatory markers. LPS treatment supported fibrinolysis, in contrast fibrinogen and thrombin decreased fibrinolytic potential of the fibroblast-like synoviocytes at mRNA level. Initiation of haemostasis in synoviocytes was affected in opposing directions by LPS and fibrinogen, shown as decreased and increased gene expression of TF, respectively. The perspectives of these findings are a deeper understanding of events taking place in an inflamed joint. Further studies with a closer clinical application are highly needed to elucidate whether haemostasis plays a role in equine joint inflammation corresponding to the current understanding of human joint disease.

## Methods

### Sample population

Synovial membranes were collected from the dorsal and palmar joint recesses of both metacarpo-phalangeal joints of six skeletally mature (10 - 13 years old) horses of different breeds euthanized for non-orthopaedic reasons at the department of Veterinary Clinical and Animal Science, University of Copenhagen according to regulations of Danish law. Samples were harvested aseptically within 1 h of euthanasia. The joints were macroscopically normal and synovial fluid appeared normal in quantity, viscosity and colour. Synovial tissue was pooled from both metacarpo-phalangeal joints of the same horse.

### Isolation and culture of equine fibroblast-like synoviocytes

Synoviocytes were isolated from the synovial tissue by enzymatic digestion (1 mg/ml collagenase type 1 (Invitrogen^TM^, Life Technologies, Nærum, Denmark), 1 % penicillin/streptomycin (Invitrogen^TM^), 1 % gentamycin (Invitrogen^TM^) in Dulbecco’s Modified Eagle Medium (DMEM) High Glucose (Invitrogen^TM^) for 3 h at 37 °C/30 rpm. The released synoviocytes were centrifuged at 450 x g for 5 min, washed twice in sterile phosphate buffered saline and cryopreserved with 10 % dimethyl sulfoxide in foetal calf serum in liquid nitrogen until study start.

The culture medium consisted of DMEM with addition of 10 % foetal calf serum (Invitrogen^TM^), 5 ml penicillin (100 IU/ml)/streptomycin (100 μg/ml), and 2.5 ml gentamycin (10 μg/ml).

At study start, the synoviocytes were thawed rapidly at 37 °C. After equalizing the cryopreserved synoviocytes with culture medium, synoviocytes were rinsed twice with culture medium to remove dimethyl sulfoxide remnants. Cells were seeded in monolayer cultures and cultured at 37 °C/5 % CO_2_ in a humidified atmosphere (passage 1). Culture medium was aspirated after 24 h to remove non-adherent synoviocytes. Culture medium was subsequently changed every 48 to 72 h until synoviocytes reached confluence, and upon confluence the synoviocytes were passaged using 0.25 % trypsin/1 mM EDTA (Invitrogen^TM^). Passage-4 synoviocytes were transferred to 24-well culture plates at a density of 250,000 cells/well. Twenty-four hours prior to stimulation the culture conditions were changed to serum-free.

### Experimental design

Initial experiments were conducted to establish concentration and incubation time for the three stimulants: LPS from *Escherichia coli* strain 055:B5 (# L2880, Sigma-Aldrich Denmark ApS), plasminogen-depleted fibrinogen (# 341578, Calbiochem, Merck, Darmstadt, Germany) and thrombin (# T7572, Sigma-Aldrich Denmark ApS) (data not shown). LPS was reconstituted in sterile water and mixed with serum-free medium and added directly to the wells in a concentration of 0.1 μg/ml as previously described [70, 71]. Fibrinogen was reconstituted in 37 °C serum-free culture medium and added directly to the wells in a concentration of 5 mg/ml as previously described [11, 25, 30]. Thrombin was reconstituted in sterile water containing 0.1 % (w/v) bovine serum albumin, mixed with serum-free culture medium and added directly to the wells in a concentration of 5 U/ml [14, 54]. Synoviocytes were harvested after 0, 6, 24, and 48 h of stimulation. Non-treated controls were cultured in standard serum-free culture medium as described above. All experiments were performed in duplicate for each horse.

### RNA isolation and quantitative real time reverse transcriptase PCR analysis

Total RNA was extracted from the synoviocytes using the Qiagen RNeasy Plus mini kit (Qiagen Nordic, Copenhagen Ø, Denmark ). Cells were first homogenized through a QIAshredder column for 2 min at 10,000 x g, and all subsequent steps were performed as described in the manufacturer’s manual. Resulting total RNA was quantified by optical density measurement (NanoDrop TM Spectrophotometer (Thermo Scientific, Wilmington, US)). Twelve randomly selected samples were run in a bioanalyzer (Agilent 2100 Bioanalyzer (Agilent Technologies, Hørsholm, Denmark)) to verify RNA quality. Total RNA isolates were kept at -80 °C until further analysis.

cDNA was synthesized from 200 ng total RNA. The reverse transcriptase PCR mastermix (Promega Biotech AS, Nacka, Sweden) consisted of 5 μL RT buffer, 1.3 μL dNTP mix (10 μM), 0.25 μL random hexamer primers (2 μg/μL), 0.25 μL Oligo-dT primers (0.5 μg/μL), 0.8 μL RNasin® Plus RNase inhibitor (40 U/μL), 1 μL M-MLV Reverse Transcriptase (200 U/μL) and water. Reverse transcription was performed in a BIOmetra® T-Gradient thermocycler (Thermo scientific, Fisher Scientific, Denmark) at 25 °C for 10 min, 42 °C for 60 min, and 95 °C for 5 min. Samples were stored at -20 °C.

Species-specific intron-spanning equine primers were used to amplify SAA, IL-6, MCP-1, TF, uPA, PAI-1, VEGF and PAR-1, and products were verified by gel electrophoresis and sequencing. Primers are listed in Table 1. Quantitative real time reverse transcriptase PCR was performed in triplicates using the LightCycler® Fast Start DNA Master SYBR Green I and LightCycler® Real-Time PCR System (Roche, Hvidovre, Denmark).

**Table 1:** Equine specific primers used for quantitative real-time reverse transcriptase PCR

Primer	Primer sequence (5′ → 3′)	Product	NCBI source/
name	Forward/reverse	size	Reference
SAA	CCT GGG CTG CTA AAG TCA TC/	169 bp	AF240364.1
	AGG CCA TGA GGT CTG AAG TG		
IL-6	ATG GCA GAA AAA GAC GGA TG/	220 bp	[72]
	GGG TCA GGG GTG GTT ACT TC		
MCP-1	ATT GGC CAA GGA GAT CTG TG/	166 bp	EU779497.1
	ATA TCA GGG GGC ATT TAG GG		
TF	TGC ACT AGC CAA CAC AAA GC/	101 bp	XM_001491449.2
	CAG AGA CAC AGC CAG GAT GA		
uPA	ATG TAT GGT GAT GCC CGT TT/	175 bp	XM_001502951.3
	CAC AGC ATT TTG GTG GTG AC		
PAI-1	AAG GGT CCG CTT CCT ACA AT/	204 bp	XM_001492517.3
	TTG AAC TGC ATT GCC TCT TG		
VEGF	CAA CGA CGA GGG CCT AGA GT/	100 bp	[73]
	CAT CTC TCC TAT GTG TGG CTT TG		
PAR1	TTC GTG ATA AGC CTG CCT CT/	212 bp	XM_001503957.2
	GTA AAA CGC TGC AGT GAC GA		
GAPDH	GGG TGG AGC CAA AAG GGT CAT CAT/	417 bp	[74]
	AGC TTT CTC CAG GCG GCA GGT CAG		

Primers used to amplify specific genes in LPS, fibrinogen, thrombin and non-treated equine fibroblast-like synoviocytes: SAA: serum amyloid A. IL-6: interleukin-6. MCP-1: monocyte chemotactic protein-1. TF: tissue factor. uPA: urokinase plasminogen activator. PAI-1: plasminogen activator inhibitor-1. VEGF: vascular endothelial growth factor. PAR-1: protease activator receptor-1. GAPDH: gluceraldehyde‐3‐phosphate dehydrogenase

### Data analysis

Results were calculated using the efficiency corrected calculation method also known as the Roche Applied Sciences E(efficiency)-method:

Normalized relative ratio (NRR) = E_t_^CT (target calibrator) – CT (target sample)^ / E_r_^CT (reference calibrator) – CT (reference sample)^ [75].

All results were normalized to reference gene gluceraldehyde‐3‐phosphate dehydrogenase (GAPDH), selected after initial testing of three reference genes (GAPDH, β-actin and ribosomal RNA (18 S)).

mRNA expression for each of the outcome parameters was compared among treatments at the different time points using a random intercept, random slope model in R [76]. For each of the outcome parameters, the NRR was log-transformed to achieve residuals (ε) which could be deemed independent identically distributed Normal (0, σ2) except for the parameter PAR-1, where a square root transformation was used and for uPA, which was not transformed at all. The general model used was thus: transformation(NRR) = α + TX + time + TX x time + ε.

Where transformation(NRR) was the transformed NRR for the specific outcome parameter (using the transformations stated above), TX the fixed and random effect of treatment (non-treated, fibrinogen, thrombin or LPS treated), time the fixed and random effect of time, and TX x time the interaction between time and TX. Statistical significance was defined as p < 0.05 for all the analyses. The results were presented as pairwise comparisons between treatments and non-treated within a time point (6, 24 and 48 h) and all measurements were compared to time point 0 h.

## Abbreviations

SAA: Serum amyloid A
TF: Tissue factor
uPA: Urokinase plasminogen activator
PAI-1: Plasminogen activator inhibitor-1
VEGF: Vascular endothelial growth factor
PAR-1: Protease activator receptor 1
MCP-1: Monocyte chemotactic protein-1
IL-6: Interleukin-6
IL-1β: Interleukin-1β
qRT-PCR: Quantitative real time reverse transcriptase polymerase chain reaction
LPS: Lipopolysaccharide
TNF-α: Tumour necrosis factor α
NRR: Normalized relative ratio
GAPDH: Glyceraldehyde-3-phosphate dehydrogenase
DMEM: Dulbecco’s Modified Eagle Medium
RNA: Ribonucleic acid
mRNA: Messenger ribonucleic acid
cDNA: Complementary deoxyribonucleic acid
EDTA: Ethylenediaminetetraacetic acid
OA: Osteoarthritis
RA: Rheumatoid arthritis
ICAM: Intercellular adhesion molecules
IL-8: Interleukin-8
